# Parent/caregiver health literacy among children with special health care needs: a systematic review of the literature

**DOI:** 10.1186/s12887-015-0412-x

**Published:** 2015-08-05

**Authors:** Jessica Keim-Malpass, Lisa C. Letzkus, Christine Kennedy

**Affiliations:** University of Virginia School of Nursing, P.O. Box 800782, Charlottesville, 22908 VA USA; University of Virginia Children’s Hospital, Charlottesville, VA USA

## Abstract

**Background:**

Children with special health care needs (CSHCN) are children with medical or behavioral diagnoses that require services beyond those generally needed by pediatric populations. They account for a significant portion of pediatric health care expenditures and often have complicated treatment regiments. Health literacy has recently been recognized as a key indicator of quality chronic disease self-management and parental/caregiver health literacy of CSHCN is an understudied area. The purpose of this systematic review was to assess the available evidence of studies investigating parent/caregiver health literacy of CSHCN.

**Methods:**

Databases were searched to retrieve relevant articles for inclusion (dating from 1998 to 2014). Only studies that assessed the relationship between parent/caregiver health literacy on outcomes pertinent to CSHCN were included. Because of the limited number of studies, there were no restrictions placed on type of outcome.

**Results:**

Thirteen studies were included in the final review with a range of health literacy assessments and outcome ascertainment. The majority of studies; (1) focused on the relationship between parental/caregiver health literacy and asthma outcomes, (2) were cross-sectional study designs, and (3) included samples recruited from pediatric clinics in academic medical settings.

**Conclusions:**

There were several gaps in the literature where future research is needed including; (1) direct assessment of child/adolescent health literacy, (2) inclusion of children with co-morbid conditions, (3) further assessment of the relationship between health literacy and health care utilization and cost, and (4) assessment of parental/caregiver health literacy in the inpatient care setting.

## Background

Children with special health care needs (CSHCN) are defined by the Maternal and Child Health Bureau as those who have or are at increased risk for a chronic physical, developmental, behavioral or emotional condition(s) and require healthcare services beyond that required by children generally [1, 2]. It is estimated that between 13-18 % of children in the United States are CSHCN and their diagnoses can encompass a wide range of pediatric diagnoses, including: autism, asthma, diabetes, sickle cell disease, cystic fibrosis, cerebral palsy, congenital heart disease, developmental delay, diseases due to prematurity, etc. [1, 3]. Among children’s health expenditures, CSHCN account for between 40 to 70 % of all health care expenditures among children [1, 3–6]. CSHCN often have multiple providers, complex treatment regimens, and high financial burden to the family, including out-of-pocket costs [7]. Additionally, they are a particularly vulnerable patient population due to the fact that they encompass a heterogeneous diagnostic group, often with multiple co-morbidities. Because their classification is not disease-specific, they are commonly encountered in health care settings, yet overlooked in terms of research and translational implications.

In adults, limited health literacy (the ability to obtain, integrate and appraise health-related knowledge) has emerged as a key indicator of adverse health outcomes including: increased measures of morbidity, poor adherence to medications, limited levels of shared decision-making, more unintended readmissions and higher utilization of health care resources compared to those with functional health literacy [8]. Health literacy is recognized by the Institute of Medicine as an integral component of high quality health care and it is estimated that nearly 36 % of adults in the United States have limited health literacy, and the prevalence rises to closer to 50 % among those from low-income backgrounds [9, 10].

While the concept of health literacy has received attention in adult populations with chronic disease, it has been substantially less studied among parents and caregivers of children. When it has been studied among parents and caregivers of children, it’s been primarily limited to health promotion, delivery of health information of well-child practices (e.g., immunization information), medication knowledge (e.g., dosing of acetaminophen), and health literacy of parents presenting to the emergency department (ED) [11–13]. There have been comparatively few studies that assess parental/caregiver health literacy of CSHCN, even though they may be at particular risk for inadequate information exchange, confusion regarding complex home medication regimens and treatment instructions, and non-adherence to medication or recommendations. Additionally, because of the chronicity of their diagnoses, it is particularly important to assess parent/caregiver health literacy across the treatment continuum. This gap in assessment and knowledge of the impact of parental/caregiver health literacy on health outcomes of CSHCN is particularly concerning and a needed area of future research. Therefore, the purpose of this systematic literature review was to assess the available evidence of studies investigating parent/caregiver health literacy of CSHCN and the effect on pertinent health outcomes.

## Methods

This systematic review was conducted using the available biomedical literature in accordance with the guidelines outlined by the Preferred Reporting Items for Systematic Reviews and Meta-Analyses Statement (PRISMA) [14].

### Data search

PubMed, OVID Medline and CINAHL were searched in October 2014 for peer-reviewed original research published prior to that date. The search strategy included: a combination of MeSH terms and key word searches including the terms: pediatric, child, parent, caregiver, health literacy, children with special health care needs, chronic disease. To ensure all relevant articles were captured, bibliographies of included articles were also reviewed for inclusion of any additional articles.

### Eligibility criteria

Prospective, retrospective and cross-sectional studies of parent/caregiver health literacy among CSHCN published between 1980 and October 2014 were considered and inclusion criteria was developed a-priori. Studies were included if they: (1) assessed parent or caregiver health literacy using a validated health literacy instrument; (2) examined the relation of parent/caregiver health literacy as an independent variable to at least one outcome variable; (3) sample included parents/caregivers of CSHCN, which includes all chronic or behavioral diseases diagnosed in childhood. Due to the low volume of health literacy studies that include CSHCN (*n* = 13), there were no restrictions placed on outcome measure. Studies were excluded if: (1) they involved a proxy for health literacy such as parent/caregiver level of education or knowledge level but did not actually assess health literacy and (2) were written in languages other than English.

### Study selection

**Fig. 1:**
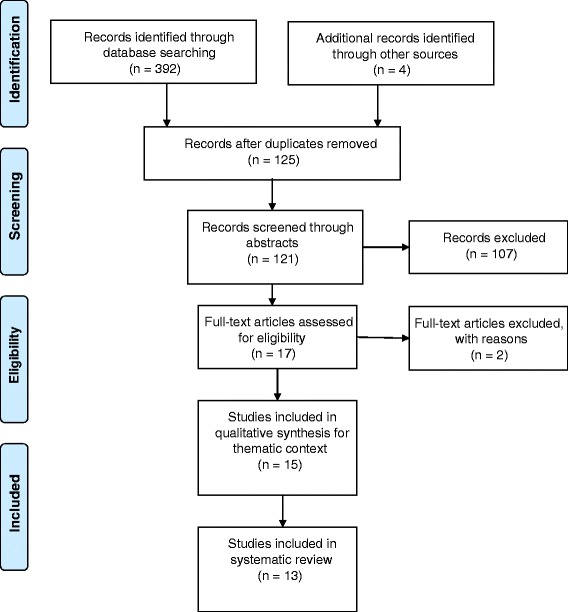
PRISMA search strategy [14]

The PRISMA flow diagram detailing study selection and inclusion is found in Fig. 1. All studies were independently assessed for eligibility by two reviewers (JKM and LL). Once articles were identified from the database searches, duplicates were removed, then all remaining abstracts were reviewed for eligibility. Full-text articles were assessed for eligibility with only two articles excluded at this stage (because there was no assessment of parent/caregiver health literacy). Fifteen studies were included for thematic and contextual purposes, and of these, thirteen studies were included in the final analysis and systematic review. Two studies were eliminated because they were reviews and not empirical studies.

### Data extraction and quality review process

A data extraction instrument was developed based on elements important to both health literacy and outcomes pertinent to CSHCN. The following criteria was collected by two reviewers (JKM and LL): author/year, study design, disease of focus, sample ascertainment, assessment of health literacy (measure), percentage of population who had limited/low/marginal health literacy, outcome measures, notable findings (that were health-literacy specific) and the study quality score. To aid in interpretation of included studies, a quality review score was used to assign a study design level and ranking criteria based on the consistency and generalizability of reported elements (quality review criteria found in Table 1). This quality review criteria was used because it does not penalize studies for being observational, and a study can still yield high, good, or low quality based on the consistency, generalizability, and potential impact of the individual study on the systematic review. The review criteria utilizes a number (based on study design) and A, B, or C (high, good, low study quality). A low study quality ranking (“C”) does not mean a poorly conducted study, it only means that there is limited ability for the authors to use the findings of that study for overall interpretation in this systematic review. The reviewers assigned quality scores independently and if there was a discrepancy, met to discuss the study to agree on a consensus score between the two reviewers.

**Table 1:** Criteria for study quality review

LEVEL I – Randomized control trial (RCT) or experimental study
LEVEL II – Quasi-experimental (no manipulation of independent variable, may have random assignment or control)
LEVEL III – Non-experimental (no manipulation of independent variable, includes descriptive, comparative, correlational studies or uses secondary data)
LEVEL IV – Qualitative (focus groups, starting point where no previous data exists).
X – Study did not meet final inclusion criteria
A - HIGH
Consistent, generalizable results
Sufficient sample size
Adequate control
Definitive conclusions
Consistent recommendations based on comprehensive literature review that includes thorough reference to scientific evidence
B - GOOD
Reasonably consistent results
Sufficient sample size for the study design
Some control
Fairly definitive conclusions
Reasonably consistent recommendations based on fairly comprehensive literature review that includes some reference to scientific evidence
C - LOW
Little evidence with inconsistent results
Insufficient sample size for the study design
Conclusions cannot be drawn

Adapted with permission from Association of Perioperative Registered Nurses (AORN) (non-published) and developed by Elayne K. Phillips, PhD, RN, FAAN and Jessica Keim-Malpass, PhD, RN

## Results

### Study characteristics

Thirteen studies meeting inclusion criteria were included in this review and study elements are described in Table 2 [15–27]. All of the studies were conducted in various settings within the United States, with the majority occurring in pediatric clinics associated with academic medical settings. The majority of studies (*n* = 7) assessed parents with much of their samples comprised of mothers, but several studies also included the term ‘caregiver’ to be inclusive of non-parental primary caregivers caring for the child (including parents, guardians, grandparents, etc.). In terms of the health literacy assessment, six studies used the Rapid Estimate of Adult Literacy in Medicine (REALM) [28], six studies used either the full or shortened Test of Functional Health Literacy in Adults (TOFHLA or S-TOFHLA) [29], two studies used the Newest Vital Sign (NVS) [30], one study used the Parental Diabetes Numeracy Test (PDNT) [31] to assess numeracy. Several studies used a combination of measures. Two studies did not provide percentages of those in their sample with limited or marginal health literacy [17, 24] and in another study all caregivers were screened as having adequate health literacy [21], which limited the interpretation of the findings in these studies for specific CSHCN populations. For the remainder of the studies, the percentage of the sample with low, limited, or marginal health literacy ranged from 5.6 % [23] to 49 % [16]. Farber and colleagues [16] who cited 49 % of their population had limited health literacy, recruited caregivers of children with asthma from an inner-city ED. Wood and colleagues [27] cited 44.4 % with a possibility of limited health literacy, specifically recruited African-American caregivers of children with asthma. In both of these cases, the confluence of race, geography, and income could explain higher sample estimates of limited health literacy than the general population, but are extremely important for the context of specific sub-populations who are at a greater risk for exhibiting limited health literacy.

**Table 2:** Included studies in systematic review of parent/caregiver health literacy of CSHCN

Author, year	Design	Disease/Sample	Assessment of HL	Outcomes	Notable findings (HL-specific)	Quality assess-ment
DeWalt et al., 2007 [15]	Retrospec-tive cohort	Asthma	REALM	ED visits	Children from parents with low health literacy had greater incidence of ED visits (IRR 1.4; 0.97-2.0), hospitalizations (IRR 4.6; 1.8-12) and days missed from school (IRR 2.8; 2.3-3.4) even after adjusting for asthma-related knowledge, disease severity, medication use, and other socio-demographic factors	IIIA
		*N* = 150	24 % of parents had low HL	Hospitalizations		
		Children (age 3–12 years) and parents from University pediatric clinic, USA		Days missed from school		
Farber et al., 1998 [16]	Cross-sectional survey	Asthma	REALM	Asthma care practices, knowledge asthma medications, management plans, prior hospitalizations and previous ED visits for asthma	All descriptive findings on frequency of asthma exacerbations and practices; not correlated with HL levels so HL interpretation limited. Not one participant had a written self-management plan.	IIIC
		*N* = 46	49 % of adults had HL 8th grade level or below; 20 % of adults had lower than 6th grade HL (low HL)			
		Children (age 2–6 years) and adults accompanying them in an inner-city emergency department (mother in 91 % of cases), all adults were African-American, USA				
Freedman et al., 2008 [17]	Prospec-tive observa-tional	Glaucoma	REALM	Adherence to eye drops, dosing errors, proportion of doses taken on schedule	Decreased parental health literacy associated with decreased medication adherence in multivariable regression model (*p* = 0.01)	IIIB
		*N* = 46	Overall HL assessments not provided			
		Children (age 5–17 years) and parents (majority mothers, percent not specified) from an academic pediatric ophthalmology clinic, USA				
Gandhi et al., 2013 [18]	Cross-sectional survey	Asthma	S-TOFHLA	Asthma control, asthma-specific HRQoL	HL-related path analysis (from HL to perceived self-efficacy with patient-physician interaction to asthma control and asthma-specific HRQoL) not statistically significant. Parents with higher HL and greater self-efficacy with patient-physician interaction had higher satisfaction with shared decision making (*β* = 0.38, *p* < 0.05)	IIIB
		*N* = 160	6.26 % of parents had inadequate or marginal HL			
		Children (age 8–17 years) and their parents (91 % female parent/guardian) from academic pediatric clinic, USA				
Harrington et al., 2013 [19]	Cross-sectional survey	Asthma	REALM and TOFHLA	Provider estimates of parental HL; perceptions influence on treatment recommendations	Providers perceptions of HL influenced asthma treatment recommendations (*p* = 0.001) and how treatment instructions were given (*p* = 0.001). Pediatric providers had low concordance between perceptions and actual parental assessment of HL.	IIIA
		*N* = 281; 14				
		Children (age 6–12 years) and their parents; 14 providers from pediatric clinic, USA	35 % of parents had either marginal or inadequate HL			
Hassan et al., 2010 [20]	Cross-sectional survey	Type 1 Diabetes	NVS	Glycemic control via mean hemoglobin A1c (HbA1c)	After controlling for race, language, income, education there was a significant relationship between HL and glycemic control (p, 0.004; R^2^ 0.23)	IIIA
		*N* = 200	17 % of parents had limited or possibly limited HL			
		Children (diagnosed at least 1 year prior) and their caregivers from academic pediatric clinic, USA				
Janisse et al., 2010 [21]	Cross-sectional survey	Type 1 Diabetes	S-TOFHLA	Glycemic control via mean hemoglobin A1c (HbA1c); Diabetes Management Scale (DMS)	HL not significantly related to DMS or HbA1c for total sample. For adolescents on intensive insulin regimen (*n* = 65), parental HL correlated with DMS adherence (*p* < 0.01).	IIIC
		*N* = 93	All caregivers screened as adequate HL			
		Adolescents (age 10–17 years) in poor metabolic control and their primary caregivers (89 % female) from a pediatric clinic, USA				
Macy et al., 2011 [22]	RCT	Asthma	REALM	Asthma knowledge	Randomized to either video (intervention) or written materials (control). Among low HL parents, improvement in knowledge regardless of education type (*p* < 0.001)	IB
		*N* = 129	31 % of parents had low HL			
		Children (age 2–14 years) with parents who presented to the ED, USA				
Porter et al., 2012 [23]	RCT	ADHD	TOFHLA	Report of sufficient and accurate clinical data	Randomized to either paper-based or computer-based data collection. Parents with adequate HL had increased odds of reporting sufficient and accurate data (sufficiency for ADHD screening: OR 8.0; 2.0-32.1; accuracy of medication report OR: 4.4; 0.5-37.4)	IB
		*N* = 182	5.6 % of parents had inadequate or marginal HL			
		Children (age 5–12 years) with parents (86 % female) from advertisement in a city, USA				
		English and Spanish-speaking participants				
Pulgaron et al., 2014 [24]	Cross-sectional survey	Type 1 Diabetes	S-TOFHLA	Glycemic control via mean hemoglobin A1c (HbA1c)	Parental numeracy and HL positively correlated (*r* = 0.37, *p* = 0.02). Parent numeracy (*r* = −0.52, *p* <0.01,), but not HL (*r* = −0.25, *p* = NS) were inversely correlated to their child’s HbA1c.	IIIB
		*N* = 70	PDNT (numeracy)			
		Children (age 3–9 years) with caregivers (84 % mothers) from diabetes clinics, USA	Overall HL assessments not provided			
		English and Spanish				
Shone et al., 2009 [25]	Cross-sectional survey	Asthma	REALM	Number of symptom free days over 2 weeks; use of urgent care in the past year; parent experiences with filling out medical forms; parent perception of asthma control; HRQoL using PACQLQ	Low parental HL was independently associated with perceiving child’s health as fair/poor (OR 3.96; 2.4-6.4), greater parent worry (OR 1.85; 1.2-2.8), needing help to read forms (OR 2.03; 1.3-3.1) and lower HRQoL (*β* = −0.097; *p* = 0.047). Measures of health care use were not associated with parent HL.	IIIA
		*N* = 499	33 % of parents had low HL			
		Children age (3–10 years) with persistent asthma and parents from an urban school district, USA				
Wittich et al., 2007 [26]	Cross-sectional survey	Asthma	TOFHLA	Provider perception of parental HL	Moderate agreement between provider perception of caregiver HL (kappa = 0.5095). Inadequately assessed HL for 16 % of caregivers.	IIIC
		*N* = 51	14 % of caregivers had inadequate or marginal HL			
		Adult caregivers (96 % female; 86 % mothers) of pediatric patients from a university-asthma clinic, USA				
Wood et al., 2010 [27]	Cross-sectional survey	Asthma	NVS	Perceived self-efficacy to manage their child’s asthma; frequency of physician visits, visits to ED, number of times admitted to hospital for asthma; asthma control	Significant relationship between HL and perceived self-efficacy to manage asthma symptoms (*r* = 0.155, *r* ^*2*^ = 0.02). There were no significant differences in HL by utilization variables or asthma control.	IIIB
		*N* = 196	44.4 % possibility of limited HL; 20.8 % high likelihood of limited HL			
		African-American children (age 5–12 years) with caregivers (84 % mothers) in urban pulmonology clinics, USA				

*ADHD* attention deficit hyperactivity disorder, *ED* emergency department, *HL* health literacy, *HRQoL* health related quality of life, *NS* non-significant, *NVS* Newest Vital Sign, *PACQLQ* Pediatric Asthma Caregiver’s Quality of Life Questionnaire, *PDNT* Parental Diabetes Numeracy Test, *REALM* Rapid Estimate of Adult Literacy in Medicine, *RCT* randomized control trial, *S-TOFHLA* Shortened Test of Functional Health Literacy in Adults, *TOFHLA* Test of Functional Health Literacy in Adults

### Sample size and study design

Sample size and study design were two of the components that were considered during the quality assessment period. The context of the current state of the science of health literacy and outcomes of CSHCN were also considered, as most studies (69 %) reported cross-sectional designs (*n* = 9) [16, 18–21, 24–27]. There were three cross-sectional studies that had adequate sample sizes and utilized appropriate statistical methods to control for confounding variables, that scored a IIIA on the quality scale due to their ability to generalize the findings to populations of CSHCN [19, 20, 25]. These three studies also described estimates of limited health literacy that were similar to rates described of the general population of adults. There was one study that utilized a retrospective cohort approach and it was also scored high on the quality scale (IIIA) because of the adequate sample size and sound statistical approach used to make conclusions on the relationship between health literacy and health service utilization [15]. There was one prospective observational study that had a low-moderate sample size where the authors’ were able to demonstrate an important relationship between health literacy and medication adherence, but they did not provide the percent of parents that had limited or marginal health literacy, therefore it scored lower as a IIIB [17].

There were also two randomized control trials (RCTs) that were a part of this analysis. Macy and colleagues [22] assessed health literacy of all enrollees, and randomized equally (not stratified based on health literacy) to either a video (intervention) or written materials (control). This study scored as a IB because the primary outcome was asthma knowledge (not a child health outcome related to asthma). Among parents with limited health literacy, asthma knowledge increased in both groups (*p* < 0.01) [22]. Porter and colleagues [23] assessed health literacy of all enrollees, and then randomized parents of children with attention deficit hyperactivity disorder (ADHD) into either a paper-based or computer-based data collection. Quality for this study was rated as IB primarily because of its lack of generalizability for CSHCN due to the outcome and primary findings (those with adequate health literacy report higher levels of sufficient and accurate clinical data) [23]. All of the included studies were from single-site recruitment, most often occurring from a pediatric clinic in academic health systems.

### Relationship of health literacy with CSHCN outcomes

Of the 13 studies in this review, eight assessed parents or caregivers of children with asthma [15, 16, 18, 19, 22, 25–27]. DeWalt and colleagues [15] focused on health service utilization among a parent/child with asthma dyad and demonstrated that children of parents with limited health literacy were more likely to use the ED (IRR 1.4, CI 0.97-2.0) and exhibited a statistically significant amount of hospitalizations (IRR 4.6, CI 1.8-12) and days missed from school (IRR 2.8, CI 2.3-3.4) after adjusting for asthma-related knowledge, medication use, and various socio-demographic factors (quality rating IIIA). Harrington and colleagues [19] focused on how provider perception of health literacy impacts asthma treatment recommendations (*p* = 0.001) and the way the information is delivered (*p* = 0.001) (quality rating IIIA). They also demonstrated that providers interpretation of health literacy had a low concordance with actual parental health literacy assessed through a validated measure [19]. Shone and colleagues [25] demonstrated that low health literacy was associated with perception of child’s health as fair/poor (OR 3.96, CI 2.4-6.4), greater parental worry about the condition (OR 1.85, CI 1.2-2.8), needing help to read the forms (OR 2.03, CI 1.3-3.1) and lower health related quality of life (*β* = −0.097; *p* = 0.047). They did not find any independent associations between health literacy and health care utilization (quality rating IIIA) [25].

There were three studies that focused on a population of parents/caregivers among children with Type 1 Diabetes Mellitus [20, 21, 24]. Hassan and colleagues [20] demonstrated a statistically significant relationship between caregiver health literacy and glycemic control as measured by HbA1c (p, 0.004; R^2^ 0.23) (quality rating IIIA). Alternatively, Janisse and colleagues [21] were not able to demonstrate the same results and did not find a relationship between health literacy and HbA1c. This interpretation is limited by the fact that their entire caregiver population screened as having adequate health literacy at baseline. For a sub-group of adolescents who were on intensive insulin regimens, higher levels of caregiver health literacy were associated with greater adherence to the diabetes management scale (*p* < 0.01) (quality rating IIIC) [21]. Finally, Pulgaron and colleagues [24] demonstrated no relationship between health literacy and HbA1c but did find that those parents with lower levels of numeracy had worse HbA1c outcomes (*r* = −0.52, *p* <0.01,) (quality rating IIIB).

Two additional studies reported on relationships between health literacy and other clinical diagnoses. Freedman and colleagues [17] conducted a prospective observational study with a small sample size that reported decreased parental health literacy was associated with decreased medication adherence among children with glaucoma, but this interpretation was limited in that they did not report overall percentages of limited health literacy (quality rating IIIB). Porter and colleagues [23] initiated the RCT among parents of children with ADHD, but it was not focused on ADHD-specific outcomes (quality ranking IB).

Correlates of the impact of limited parental/caregiver health literacy on child health outcomes were synthesized across all studies (Table 3). The impact of limited health literacy was assessed for utilization, disease management, communication/knowledge transfer, and information appraisal outcomes. The impact of parental/caregiver health literacy on three of the four factors (utilization, communication and knowledge transfer and information appraisal) were deemed inconclusive because of the limited number of studies that were able to establish associations. Limited health literacy was determined to have an overall mediocre association on disease management because five of the included studies established correlations between limited health literacy and poorer disease management outcomes, while two studies described non-significant findings.

**Table 3:** Correlates of lower health literacy on outcomes of CSHCN

Factor type	Factors	Association (# of studies)	Strength of finding
Utilization	ED visits	Positive (1)	Inconclusive
	Hospitalization	Neutral/non-significant (1)	
	Days missed from school		
Disease management	Medication adherence	Negative (5)	Mediocre
	Adherence to treatment recommendations	Neutral/non-significant (2)	
	Clinical labs (HbA1c control)		
	Symptom control		
	HRQoL		
Communication and knowledge transfer	Shared decision making	Negative (2)	Inconclusive
	Quality of how instructions delivered		
	Patient-physician interaction		
Information appraisal	Accuracy of medical report/history	Negative (2)	Inconclusive
	Ability to read forms		

## Discussion

The data presented in the 13 studies included in this review highlight the future directions needed in parental/caregiver health literacy among CSHCNs. While numerous studies demonstrated a relationship between parental/caregiver health literacy and outcomes pertinent to CSHCNs, several gaps in the literature remain. All studies utilized valid and reliable instruments to assess for parental/caregiver health literacy, however there was a wide range of health literacy estimates. This can likely be explained due to the heterogeneity in sampling strategies and locations. A defined need in the literature is health literacy assessment of the child/adolescent themselves – particularly among older school age and adolescent children who are likely engaging in some form of self-management of their own disease. For example, the Newest Vital Sign (NVS) has been administered in children as young as seven [32] and the Rapid Estimate of Adolescent Literacy in Medicine (REALM)-Teen was specifically validated as a version of the REALM to be used among adolescents age 10–19 years of age [33]. Very few studies have assessed adolescent health literacy. In previous research, Dharmapuri and colleagues [34] were not able relationship between adolescent health literacy and medication adherence. However, more adolescent-specific health literacy studies are needed with more diverse populations and among CSHCNs, where medication regimens are often chronic in nature, rather than episodic. There has been a limited discussion about the importance of a developmentally appropriate approach to health literacy among children and adolescents as well as the proposed importance of the health literacy of the child/parent dyad and these should be considered gaps in the current state of the literature [11].

The majority of the parental/caregiver health literacy studies included in this review focused on asthma. While asthma provides a concrete disease model for self-management of chronic disease in childhood and adolescence, more studies are needed from other representative special health care needs diagnoses. A defined need in this body of literature includes studies that encompass comprehensive symptom management approaches among families with limited health literacy. CSHCN often have multiple co-morbid conditions, and none of the included studies involved samples with more than one condition. Along with more representative CSHCN diagnoses, another defined gap is the focus of limited health literacy families in the acute or critical care inpatient hospital setting. There are no identified studies that assess parental/caregiver health literacy in this setting and there are likely many relationships between limited health literacy and communication between providers, information appraisal, and transition from hospital to the home care setting.

While there was a range in study quality, several studies included in this review demonstrated relationships between parental/caregiver health literacy and outcomes that are meaningful for families of CSHCN. Even so, very few studies actually assessed the impact of parental/caregiver health literacy on health care utilization. DeWalt and colleagues [15] found that children from parents with low health literacy with asthma had greater incidence of ED visits and hospitalizations, while Wood and colleagues [27] were not able to establish any relationship between caregiver health literacy on utilization variables related to asthma visits. The commonly proposed pathway of limited health literacy inhibiting medication adherence, which ultimately may cause an increase in overall emergent, ED and hospital utilization exits, alternative causal mechanisms must also be considered. Additionally, no studies assessed the relationship of parental/caregiver health literacy on identification of early warning signs of uncontrolled symptoms or disease progression, and these concepts are incredibly central to self-management of CSHCN in the home setting, which may also impact utilization. Finally, no studies included in this review assessed the impact of health literacy on cost, cost-savings, or cost-utility of chronic disease management. The relationship between health literacy and the direct financial impact on families, payers, and health systems are not yet fully understood, particularly among the pediatric population.

Very few studies included in this review appropriately considered the confluence of health literacy among other social determinants of health that may lead to adverse health outcomes for CSHCN. Sanders and colleagues have described that due to the dependence of children on many caregivers, researchers in pediatric health literacy may want to consider the collective health literacy of all responsible for the care of that child, including the child, parent, siblings, family members, daycare or school staff, etc. [11]. Collective health literacy of the child may be operationalized as a form of social capital and has broad implications for clinical and research outcomes [11].

### Limitations

Most of the studies included in this review were observational studies, which leaves room for bias in interpretation of the results. To attempt to offset this bias, we utilized review criteria to help the authors’ interpret the strength of the study in terms of the generalizability and implications for CSHCN. Additionally, because we wanted to understand the impact of parental/caregiver health literacy on a wide range of child health outcomes we included all diagnoses that would classify the children as CSHCN. The heterogeneity in disease samples may limit the ability to interpret our results. Finally, we did not include child health outcomes related to healthy children, health knowledge or health promotion strategies. The vast majority of parent/caregiver health literacy research to-date includes healthy children so there may be important relationships we were not able to fully describe due to this exclusion.

## Conclusions

Assessing for and addressing limited health literacy is a critical component of future research endeavors aimed at improving child health outcomes and health care utilization for vulnerable families. This review of the literature highlights early key concepts that must be further developed into more robust prospective studies as well as intervention-development and testing through RCTs. Interdisciplinary teams are needed to develop innovative modalities to integrate this research into current clinical practice. Finally, clinicians and researchers must work together with policy-makers to advocate for the health of limited health literacy families.

## Abbreviations

ADHD: Attention deficit hyperactivity disorder
CSHCN: Children with Special Health Care Needs
ED: Emergency department
HL: Health literacy
HRQoL: Health related quality of life
NS: Non-significant
NVS: Newest vital sign
PACQLQ: Pediatric asthma caregiver’s quality of life questionnaire
PDNT: Parental diabetes numeracy test
PRISMA: Preferred reporting items for systematic reviews and meta-analyses statement
REALM: Rapid estimate of adult literacy in medicine
RCT: Randomized control trial
S-TOFHLA: Shortened test of functional health literacy in adults
TOFHLA: Test of functional health literacy in adults
